# Development, Prevention, and Treatment of Alcohol-Induced Organ Injury: The Role of Nutrition

**DOI:** 10.35946/arcr.v38.2.11

**Published:** 2017

**Authors:** Shirish Barve, Shao-Yu Chen, Irina Kirpich, Walter H. Watson, Craig McClain

**Affiliations:** Shirish Barve, Ph.D., is a Professor in the Division of Gastroenterology, Hepatology, and Nutrition, Department of Medicine, and a Professor in the Department of Pharmacology and Toxicology; Shao-Yu Chen, Ph.D., is a Professor in the Department of Pharmacology and Toxicology; Irina Kirpich, Ph.D., and Walter H. Watson, Ph.D., both are Assistant Professors in the Division of Gastroenterology, Hepatology, and Nutrition, Department of Medicine, and in the Department of Pharmacology and Toxicology; all at the University of Louisville School of Medicine, Louisville, Kentucky. Craig McClain, M.D., is a Professor in the Division of Gastroenterology, Hepatology, and Nutrition, Department of Medicine, and a Professor in the Department of Pharmacology and Toxicology at the University of Louisville School of Medicine, Louisville, Kentucky, and a Staff Physician at the Robley Rex Veterans Medical Center, Louisville, Kentucky

**Keywords:** Alcohol consumption, alcohol use, abuse, and disorder, heavy alcohol consumption, alcohol-nutrition interactions, organ injury, tissue injury, intestine, nutrition, nutrients

## Abstract

Alcohol and nutrition have the potential to interact at multiple levels. For example, heavy alcohol consumption can interfere with normal nutrition, resulting in overall malnutrition or in deficiencies of important micronutrients, such as zinc, by reducing their absorption or increasing their loss. Interactions between alcohol consumption and nutrition also can affect epigenetic regulation of gene expression by influencing multiple regulatory mechanisms, including methylation and acetylation of histone proteins and DNA. These effects may contribute to alcohol-related organ or tissue injury. The impact of alcohol–nutrition interactions has been assessed for several organs and tissues, including the intestine, where heavy alcohol use can increase intestinal permeability, and the liver, where the degree of malnutrition can be associated with the severity of liver injury and liver disease. Alcohol–nutrition interactions also play a role in alcohol-related lung injury, brain injury, and immune dysfunction. Therefore, treatment involving nutrient supplementation (e.g., with zinc or S-adenosylmethionine) may help prevent or attenuate some types of alcohol-induced organ damage.

The effect of alcohol on organ health and injury is complex and influenced by a host of different factors, such as dose of alcohol consumed; duration and pattern of drinking (e.g., binge drinking); and, as reviewed in this article, potential interactions with nutrition. The *2015–2020 Dietary Guidelines for Americans* (U.S. Department of Health and Human Services and U.S. Department of Agriculture 2015) highlight the concept of the standard drink and the fact that if alcohol is consumed, it should be in moderation (i.e., up to 1 drink per day for women and 2 drinks per day for men in adults of legal drinking age). It is becoming increasingly accepted that this moderate form of drinking may have health benefits that seem to lessen many types of organ injury. This concept was popularized in 1991, when Morley Safer presented information on the television show *60 Minutes* related to the “French paradox”—that is, the observation that the French seemed to have lower rates of heart attacks despite higher fat consumption. This outcome was postulated as possibly resulting from the beneficial effects of wine consumption by the French. Subsequent studies have shown that all forms of alcohol, when consumed in moderation, seem to lower the risk of coronary artery disease (Yang et al. 2016). The beneficial effect can be represented by a J-shaped curve, in which low alcohol consumption has protective effects compared with abstention, whereas excessive alcohol consumption is harmful. Moderate drinking also may have beneficial effects on several other organs and organ systems, including the following:

- Decreased risk of ischemic stroke (Sacco et al. 1999);
- Protection against type 2 diabetes (Conigrave et al. 2001);
- Decrease in rheumatoid arthritis (Di Giuseppe et al. 2012);
- Improved cognition (Anstey et al. 2009);
- Decreased progression of liver disease to fibrosis in obese individuals (Thomson et al. 2012); and
- Improved renal function (Koning et al. 2015).

Indeed, moderate alcohol consumption may be associated with an overall modest survival benefit (Ford et al. 2011).

Moderate alcohol consumption also has been shown to decrease biomarkers of inflammation, such as C-reactive protein, and reduced inflammation could be one unifying mechanism underlying alcohol’s protective effects (Imhof et al. 2004). On the other hand, long-term heavy alcohol abuse can cause organ injury, which may, at least in part, result from alcohol–nutrient interactions and alcohol-related nutrient deficiencies. As described in this article, people who abuse alcohol frequently consume large amounts of alcohol, which may contribute to the displacement of needed nutrients (see figure 1). Indeed, recent analyses of nutritional status and alcohol consumption in people with alcohol use disorder (AUD) who were admitted to a rehabilitation program demonstrated that the participants generally had a normal body mass index, were not overtly malnourished, and did not have clinical evidence of alcohol-induced organ injury. However, these people were consuming, on average, 14 drinks per day, which would amount to about 2,000 calories per day or more consumed as alcohol (Vatsalya et al. 2016). Considering that the participants had a normal body mass index, this suggests that they replaced normal nutrients with alcoholic beverages, resulting in potential nutrient deficiencies. Nutritional supplementation may either help ameliorate such deficiencies or have pharmacologic effects.

Alcohol and nutrition can interact at multiple levels. For example, alcohol metabolism can result in the generation of reactive oxygen species, which can deplete endogenous nutritional antioxidant stores and contribute to oxidative stress. Heavy alcohol consumption also can cause poor intestinal absorption of certain nutrients (e.g., zinc) or increase nutrient losses (e.g., by increasing zinc and magnesium excretion in the urine). Moreover, nutrition can have a far-reaching impact through altering epigenetic mechanisms, such as methylation and acetylation of DNA and associated proteins. Finally, the degree of alcohol-related malnutrition can be associated with the severity of organ injury (e.g., alcoholic hepatitis). This article reviews how nutritional alterations may predispose to alcohol-induced organ injury and how nutritional supplementation may prevent and/or treat alcohol-induced organ injury. The article specifically highlights the effects of certain alcohol–nutrient interactions, with a focus on zinc and linoleic acid, and their impact on epigenetics and selected organ injury.

## Nutrition and Nutritional Alterations Following Alcohol Use/Abuse

### Alcohol: Nutrition Overview

From a nutrition perspective, alcohol is a significant source of calories, but these can be considered “empty” calories—that is, they contain few micronutrients, such as vitamins and minerals, normally found in most food sources (Antonow and McClain 1985). The main site of beverage alcohol (i.e., ethanol) metabolism is the liver, where ethanol is converted to carbon dioxide and water, with an energy yield of 7 kcal/g of alcohol. Regular alcohol intake can be a major source of calories, because beer has approximately 150 kcal per 12-ounce can and bourbon or scotch with a mixer has approximately 125 kcal per drink. Thus, a person can easily consume 200 to 500 calories or more per day by consuming 2 to 3 drinks. For people attempting weight reduction, alcohol consumption therefore can be considered a source of unwanted and empty calories. Moreover, when alcohol intake is combined with fructose-containing sugared drinks, the intake of empty calories increases even further, enhancing the opportunity for alcohol-induced organ injury. Finally, alcohol can be an expensive source of calories compared with traditional foods, and this may become a major problem for people with limited incomes.

The issue of alcohol as a nutrient becomes more prominent when dealing with people with AUD and those with alcohol-induced organ injury. Analyses of the nutritional status of people with AUD admitted to treatment programs found that these individuals often consumed 35 to 50 percent of their total calories as alcohol, and some exhibited inadequate micronutrient intake and micronutrient serum levels (Antonow and McClain 1985). However, most had little or no evidence of protein-calorie malnutrition and loss of muscle mass. In contrast, patients admitted to hospitals for severe alcoholic hepatitis who also consumed 50 percent of their total calories as alcohol not only regularly showed depletion of certain micronutrients but also loss of muscle mass (Mendenhall et al. 1995*a*). The following sections focus on the micronutrient zinc, which may be deficient or have altered metabolism with heavy alcohol consumption, and a macronutrient (i.e., dietary fat) that may play a role in alcohol-induced organ injury. Some of the other micronutrients for which heavy alcohol intake may cause deficiency states or altered metabolism are listed in the table.

### Zinc

**Figure 2:**
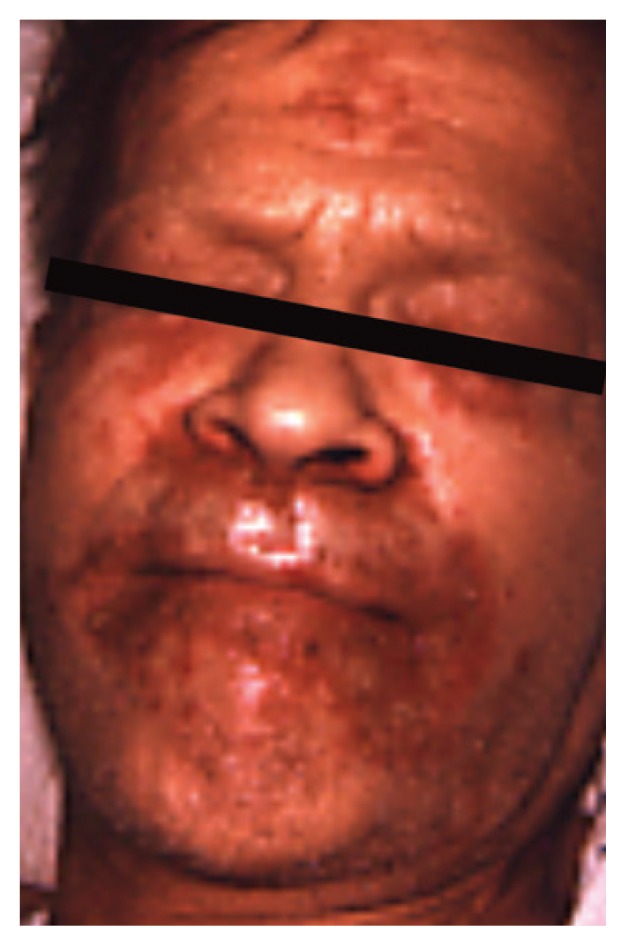
Chronic alcohol user who had been consuming large amounts of beer before admission. Note classical skin lesions of zinc deficiency around the eyes, nose, and mouth.

Zinc is an essential trace element required for normal cell growth, development, and differentiation, including such processes as DNA synthesis, RNA transcription, and cell division and activation. It is a critical component of many proteins/enzymes, including zinc-dependent transcription factors. Zinc deficiency or altered zinc metabolism is frequently observed in heavy alcohol drinkers and may result from decreased dietary intake, increased urinary excretion, abnormal activation of certain zinc transporters, and induction of hepatic metallothionein (Mohammad et al. 2012). Zinc deficiency may manifest itself in many ways in alcoholics, ranging from raised, crusting skin lesions around the eyes, nose, and mouth (figure 2) to impaired wound healing or liver regeneration, altered mental status, or altered immune function (Mohammad et al. 2012). Importantly, oxidative stress (e.g., resulting from ethanol metabolism) may cause release of zinc from critical zinc-finger proteins and cause loss of DNA-binding activity. Specifically, oxidative stress causes modification of certain amino acids (i.e., cysteine residues) that hold the zinc in place in zinc-finger proteins such as hepatocyte nuclear factor 4 (HNF4), a transcription factor that is essential for liver development.

**Figure 3:**
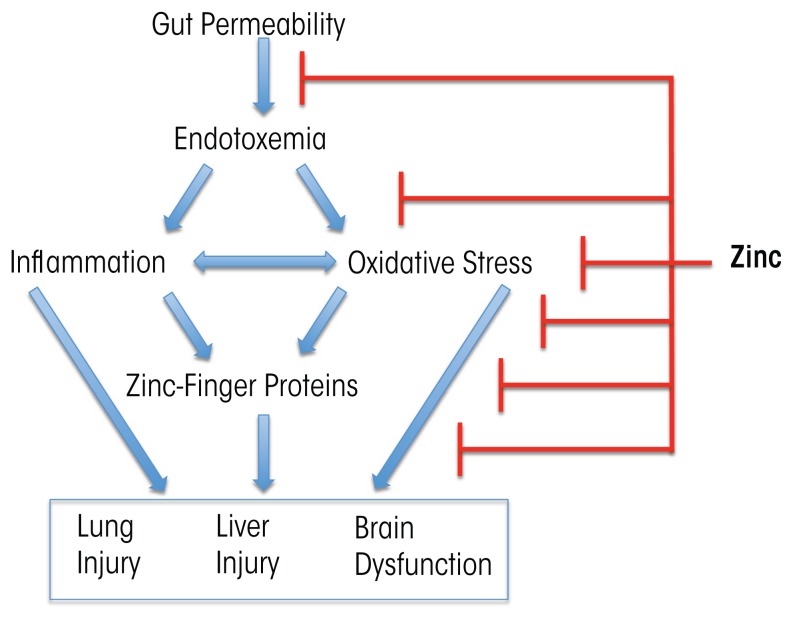
Zinc therapy positively affects multiple mechanisms of alcohol-induced organ injury. Thus, zinc enhances the gut barrier and tight junctions, thereby reducing gut permeability and the risk of transfer of bacterial endotoxin into the blood (i.e., endotoxemia). In addition, zinc decreases proinflammatory cytokine production and oxidative stress and ensures proper functioning of important zinc-dependent regulatory proteins (e.g., zinc-finger proteins). Through these and other mechanisms, zinc supplementation can improve liver injury and may attenuate lung and brain dysfunction.

Zinc supplementation has been documented to block or attenuate experimental organ injury and dysfunction in the gut, liver, lung, and brain through multiple pathways. Thus, zinc may strengthen the integrity of the intestinal wall by stabilizing tight junctions, reduce transfer of toxic bacterial molecules (e.g., endotoxin) into the blood, lower the levels of metabolic toxins such as ammonia in the blood, decrease production of inflammation-promoting (i.e., proinflammatory) cytokines, reduce oxidative stress, and attenuate apoptotic cell death (Zhong et al. 2010, 2015) (figure 3). The dose of zinc used for treatment of alcohol-induced organ injury such as liver disease usually is 50 mg of elemental zinc taken with a meal to decrease the potential side effect of nausea. Intake of greater than 50 mg of elemental zinc per day can cause dose-related side effects, such as copper deficiency resulting from reduced copper absorption.

### Dietary Fats

The critical role for specific types of dietary fat (i.e., saturated versus unsaturated fats) in intestinal and liver injury has been demonstrated and extensively studied in preclinical animal models of alcohol feeding using various sources of dietary lipids. Experimental evidence has shown that dietary saturated fats (SFs) attenuated, and unsaturated fats (USFs) enhanced, alcohol-induced liver damage (Nanji and French 1989). Thus, in contrast to the general assumption that SFs are less healthy than USFs, in this situation SFs had a protective effect and USFs had a harmful effect.

Further analyses focused on the role of different types of dietary polyunsaturated fatty acids (PUFAs) in alcohol-induced gut and liver injury. There are two major families of dietary PUFAs— omega-6 [ω-6] and omega-3 [ω-3] PUFAs—each of which includes numerous related metabolites. It has been demonstrated that linoleic acid, an ω-6 PUFA [18:2ω-6], is required for the development of experimental alcohol-induced intestinal and liver injury and that the severity of alcoholic liver disease (ALD) is correlated with the amount of linoleic acid in the diet (Nanji and French 1989; Ronis et al. 2004). Conversely, fish oil (a rich source for ω-3 PUFAs) or purified ω-3 PUFAs (e.g., eicosapentaenoic acid [EPA] and docosahexaenoic acid [DHA], which are known to be important in brain development) may be beneficial in ALD. For example, in mice, prior ingestion of fish oil, specifically tuna fish oil, in amounts that provided 30 percent of the total caloric intake, resulted in reduced hepatic fat accumulation caused by a single dose of ethanol administration. This effect was mediated, at least in part, through marked reductions in the expression of the hepatic enzyme stearoyl-CoA desaturase-1 and in the activity of the transcription factor sterol regulatory element–binding protein (Wada et al. 2008). Mice supplemented with highly purified DHA also had significantly decreased alcohol-induced liver steatosis, inflammation, and injury (Huang et al. 2013). The beneficial role of ω-3 PUFAs in experimental ALD also has been supported by the observation that when rhesus monkeys who had free access to an ethanol solution were fed a diet that was generally nutritionally adequate (including the linoleic acid amount), but with a low ω-3 PUFA content (i.e., a very low concentration of α-linolenic acid), the animals developed hepatic steatosis and fibrosis (Pawlosky and Salem 2004). The ω-3 PUFAs also are precursors to factors that resolve injury and inflammation, such as resolvins (e.g., E- and D-series resolvins generated from EPA and DHA, respectively), and a high dietary ω-6/ω-3 PUFA ratio may be disadvantageous to resolving inflammation (Serhan and Petasis 2011). Thus, emerging evidence suggests that dietary fats can play a role in both initiation and treatment of alcohol-induced organ injury in the gut and liver as well as in the brain (which will be discussed later in this article).

## Nutrition–Alcohol Interactions and Epigenetics

In virtually every cell type, epigenetic mechanisms—that is, modifications to the genetic material that do not alter the DNA sequence—play a critical role in both the physiologic and pathologic regulation of gene expression. These mechanisms, which involve chromatin remodeling initiated by posttranslational modifications of histones and changes in DNA methylation status, can activate or deactivate gene transcription. The proteins that are involved in posttranslational histone modifications and DNA methylation changes require a variety of cofactors, including acetyl coenzyme A, S-adenosylmethionine (SAM), nicotinamide adenine dinucleotide, and zinc (Moghe et al. 2011). A person’s nutritional status can significantly influence the availability of these cofactors and, consequently, epigenetic mechanisms, gene expression, and disease pathogenesis. Chronic alcohol consumption is known to affect nutritional status at many levels, including nutrient intake, absorption, utilization, and excretion, causing nutritional disturbances and deficiencies in these cofactors. Research has determined that alcohol-induced nutrient fluctuations can impact transcriptional activity and expression of genes by modulating epigenetic parameters, including histone modifications and DNA methylation (Moghe et al. 2011; Zakhari 2013). Hence, in people with AUD, the combined effects of alcohol metabolism and compromised nutrition are likely to influence epigenetic mechanisms, gene expression, and disease pathogenesis involving intestinal barrier dysfunction, immune suppression, and organ injury.

### Alcohol’s Effects on Histone Acetylation and Methylation

It is becoming increasingly evident that histone-associated epigenetic modifications, such as histone acetylation and methylation, play a significant role in the regulation of gene expression and development of alcohol-induced organ pathology, such as liver disease and immune dysfunction (Moghe et al. 2011). In particular, histone acetylation in promoter regions is a key regulator of gene expression and is associated with enhanced transcriptional activity, whereas deacetylation typically is associated with transcriptional repression. Steady-state levels of acetylation result from the balance between the opposing activities of two groups of enzymes—histone acetyltransferases and histone deacetylases. The expression and activities of both types of enzymes can be influenced by alcohol and cofactors, such as nicotinamide adenine dinucleotide and zinc (Ghare et al. 2014; Moghe et al. 2011). Taken together, epigenetic histone modifications provide a likely link between alcohol-mediated nutrient alterations in gene expression and disease pathogenesis.

### Alcohol’s Effects on DNA Methylation

Investigation of the dietary influences on epigenetic processes has revealed a direct link between SAM, which serves as the primary biological methyl donor, and DNA methylation changes that epigenetically influence gene expression (McCabe and Caudill 2005). In general, DNA hypermethylation at DNA sequences called CpG islands in gene promoters leads to transcriptional silencing, whereas DNA hypomethylation allows for transcription to occur.

Excessive alcohol consumption can decrease SAM levels via multiple mechanisms, such as reduced folate levels and inhibition of key enzymes in one-carbon metabolism. The reduced SAM levels lead to aberrant DNA methylation patterns and pathogenic alterations in gene expression (Varela-Rey et al. 2013). Importantly, alcohol-induced perturbations in global and regional DNA methylation have been linked with diverse pathological conditions, including ALD, carcinogenesis in various organs, alcohol dependence, and fetal alcohol spectrum disorders (FASD), to name only a few. Clearly, further research is needed to detail the alcohol–nutrient interactions that influence epigenetic mechanisms underlying pathogenic changes in gene expression and disease progression, with the goal of developing nutrient-based therapies.

## Examples of Nutrition–Alcohol Interactions in Alcohol-Induced Organ/Tissue Injury

### Intestine

The intestinal mucosa plays a critical role in preventing passage of toxins from the intestine into the bloodstream, as well as in immune function, detoxification, and metabolism. The importance of the gut in alcohol-mediated multiorgan pathology is becoming increasingly recognized. Clinical and experimental data have demonstrated that the gut-derived bacterial product, lipopolysaccharide, also referred to as endotoxin, plays a crucial role in the development and progression of alcohol-induced organ injuries, including ALD. Significantly increased endotoxin levels in the blood (i.e., endotoxemia) have been found in patients with different stages of ALD, including fatty liver, hepatitis, and cirrhosis (Parlesak et al. 2000).

**Figure 4:**
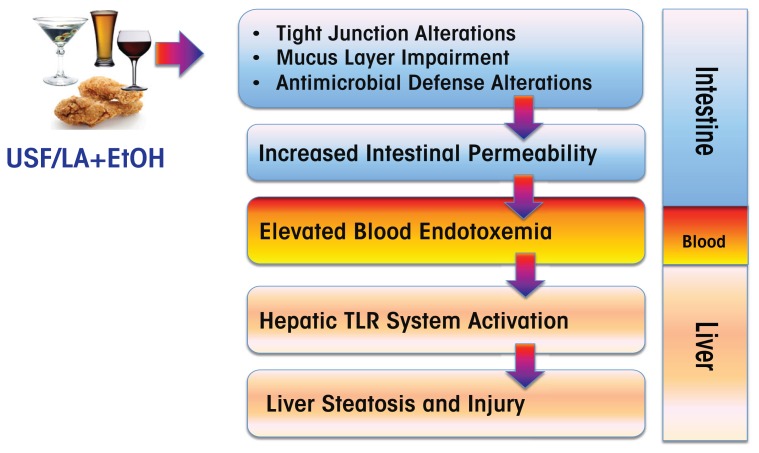
Alcohol (EtOH) consumption combined with dietary intake of unsaturated fatty acids (USFs) (e.g., linoleic acid [LA]) can have numerous deleterious effects on the intestine, blood, and liver. In the intestine, this combination changes the bacterial composition (microbiome) and interferes with various aspects of the body’s defense systems, thereby increasing intestinal permeability. This leads to endotoxemia and liver injury. NOTE: TLR = toll-like receptor.

**Figure 5:**
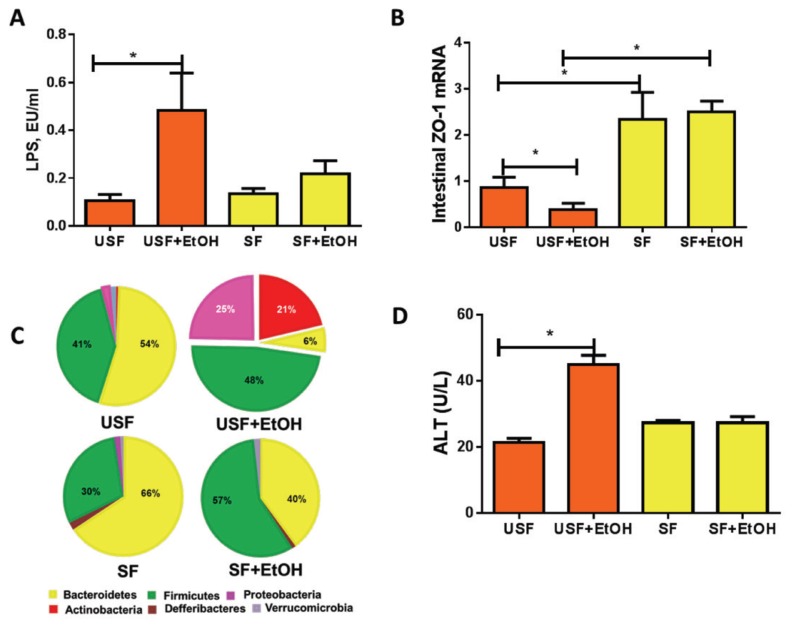
Effects of saturated fat (SF) and unsaturated fat (USF) diets on endotoxemia, intestinal tight junctions, gut microbiome, and liver injury in response to chronic alcohol (EtOH) feeding. **(A)** Plasma endotoxin levels assessed by plasma lipopolysaccharide (LPS) measurement. Alcohol feeding significantly increases LPS levels in the plasma when combined with a USF diet. **(B)** Levels of the mRNA for the tight-junction protein zonula occuldens-1 (ZO-1) in the intestine. Animals receiving a USF diet showed greater disruption of tight junctions (i.e., lower ZO-1 levels) than animals receiving a SF diet; this effect was exacerbated with alcohol feeding. **(C)** Comparative analysis of the relative abundance of different phyla of gut bacteria in mice fed ethanol and different types of dietary lipids. The phyla abundance is indicated by the color bars. **(D)** Liver injury was evaluated by plasma alanine aminotransferase (ALT) activity. In animals receiving a USF diet, but not in those receiving a SF diet, alcohol feeding caused significant liver injury. NOTE: Horizontal bars indicate statistically significant differences.

Multiple mechanisms contribute to alcohol-associated endotoxemia, including alcohol-mediated alterations in the composition of the bacterial population of the gut (i.e., gut microbiome) (Mutlu et al. 2009) and increased lipopolysaccharide translocation as a result of disruption of intestinal barrier integrity. Recent studies in mice have demonstrated that the type of dietary fat consumed can influence alcohol-induced changes in the gut microbiome composition (and, therefore, function), intestinal injury/inflammation, and intestinal barrier function (figures 4 and 5). Specifically, when comparing animals that were fed either dietary USFs or SFs plus ethanol (EtOH),^1^ the studies found the following:

- The animals that received EtOH+ USF showed increased gut permeability and elevated endotoxemia compared with those that received EtOH+SF (Kirpich et al. 2012) (figure 5A).
- Compared with EtOH+SF, a chronic EtOH+USF diet triggered an intestinal proinflammatory response characterized by increased levels of several cytokines, including tumor necrosis factor-α and monocyte chemoattractant protein-1. In addition, the intestinal mucus layer and antimicrobial defenses were altered (Kirpich et al. 2013).
- Intestinal inflammation was positively correlated with the EtOH+USF-triggered disruption of the intestinal tight junctions (figure 5B). Even in the absence of alcohol, a USF diet resulted in downregulation of intestinal expression of tight-junction protein mRNA compared with an SF diet. Alcohol further suppressed tight-junction proteins in animals receiving EtOH+USF, but did not affect intestinal tight junctions in the EtOH+SF group (Kirpich et al. 2013) (figure 5B).
- Unlike EtOH+SF, dietary EtOH+USF caused alterations in gut microbiota (Bull-Otterson et al. 2012; Kirpich et al. 2016) (figure 5C).^2^ The observed microbiota and intestinal barrier changes were associated with significant liver steatosis, inflammation, and injury in EtOH+USF-fed mice (figure 5D). These adverse effects of ethanol on the liver were markedly attenuated by a SF diet containing medium-chain triglycerides.

Thus, it is clear that the interactions of dietary fat and alcohol are important in mediating alcohol-induced intestinal and liver injury.

Similarly, in mice, zinc deficiency associated with chronic alcohol intake led to markedly decreased tight-junction proteins and increased endotoxemia. Zinc supplementation corrected these effects through multiple mechanisms, including zinc-finger function and epigenetic mechanisms (Zhong et al. 2015). In summary, an important component of alcohol-induced organ inflammation/injury arises in the gut and may be modified by nutrition.

### Liver Injury

Patients with severe alcoholic hepatitis almost invariably demonstrate some form of malnutrition. Probably the most detailed information concerning malnutrition in ALD comes from two large studies from the Veterans Health Administration (VA) Cooperative Studies Program in patients with alcoholic hepatitis (Mendenhall et al. 1984, 1986, 1995*a*,*b*). In these studies, almost 50 percent of the patients’ energy intake was derived from alcohol. Although they frequently showed no inadequate calorie intake, the patients often exhibited insufficient intake of protein and critical micronutrients. The severity of liver disease generally correlated with the severity of malnutrition. During treatment, the patients received a balanced 2,500-kcal hospital diet (monitored by a dietitian) that they were encouraged to consume. Investigators found that voluntary oral food intake correlated in a stepwise fashion with 6-month mortality data. Thus, patients who voluntarily consumed more than 3,000 kcal per day had virtually no mortality, whereas those who consumed less than 1,000 kcal per day had a 6-month mortality of more than 80 percent (Mendenhall et al. 1995*a*). Moreover, the degree of malnutrition correlated with the development of serious complications, such as encephalopathy, ascites, and hepatorenal syndrome (Mendenhall et al. 1995*a*).

Initial interest in nutrition therapy for ALD was stimulated by Patek and colleagues (1948) who demonstrated that a “nutritious diet” improved the 5-year outcome of patients with alcoholic cirrhosis compared with historic control subjects. Subsequently, nutritional supplementation through a feeding tube was shown to significantly improve liver function in in-patients with ALD compared with inpatients who ate a hospital diet (Kearns et al. 1992). Probably the most important data supporting nutrition therapy came from a multicenter study by Cabré and colleagues (2000), who randomly assigned patients with severe alcoholic hepatitis to receive either the glucocorticoid prednisone (40 mg daily) or a liver-specific formula containing 2,000 calories per day through a feeding tube.^3^ The 1-month mortality was the same in both groups, but the 1-year mortality was significantly lower in the enteral-nutrition group than in the glucocorticoid group, mainly because they experienced fewer infectious complications. This study clearly documented the importance of enteral nutrition in severe alcoholic hepatitis. Oral/enteral nutrition is preferable over parenteral nutrition because of lower costs, risk of sepsis from the parenteral nutrition line, preservation of the integrity of the gut mucosa, and prevention of bacterial translocation and multiple-organ failure.

Enteral nutrition supplements also have been shown to improve nutritional status and immune function in outpatients with alcoholic cirrhosis as well as to reduce hospitalization. The concept of an outpatient late-evening snack (prior to bedtime) was established after studies demonstrated altered energy metabolism in people with liver cirrhosis. These patients exhibit depleted hepatic glycogen stores, which force the body to depend on fat and protein stores, leading to catabolism during an overnight fast. A randomized controlled trial demonstrated that provision of a late-evening nutritional supplement (compared with daytime supplements) over a 12-month period could improve body protein stores in patients with cirrhosis. The nighttime snack resulted in body protein accrual equivalent to about 2 kg of lean tissue sustained over 12 months, whereas this benefit was not observed with daytime snacks. Thus, late-evening snacks are valuable nutritional interventions in outpatients with alcoholic cirrhosis (Plank et al. 2008).

Many types of nutritional supplements have yielded positive effects in animal models of ALD, especially antioxidants. However, human studies using specific nutrients or combination therapy are limited and generally have shown equivocal or negative results. Larger, well-designed studies are required.

### Lung Injury

Chronic alcohol abuse alters the phenotype of the lung and makes it more susceptible to subsequent challenges, such as bacterial infection and acute lung injury. One of the mechanisms that contribute to increased susceptibility to infection and injury is alcohol-induced oxidative stress. Oxidative stress is defined as an imbalance between oxidants and antioxidants, and the way cells sense and respond to such an imbalance is a key determinant of disease initiation/progression or resolution. Oxidant-sensing and -signaling pathways rely primarily on proteins with reactive thiol-containing cysteine residues. The reactivity of a given protein thiol can be fine tuned by its local redox environment—that is, by the ratio of reduced versus oxidized molecules in the cell. This redox environment largely is controlled by two low-molecular-weight thiol-disulfide redox couples: one composed of the amino acid cysteine (Cys), which is the reduced partner of the pair, and its disulfide cystine (CySS), which serves as the oxidized partner. The other redox pair comprises glutathione (GSH) as the reduced partner and its disulfide GSSG as the oxidized partner. The two pairs are related but have different roles. Cys is one of the three component amino acids making up GSH, so it is not surprising that they share similar chemical properties. However, these redox control systems are compartmentalized; GSH/GSSG provides control mechanisms within cells and in the lung-lining fluid, whereas Cys/CySS predominates in the extracellular fluids of plasma and interstitium. The extracellular Cys/CySS redox state has been shown to have a direct effect on the production of two important proinflammatory cytokines, namely production of transforming growth factor β by lung fibroblasts (Ramirez et al. 2007) and interleukin-1 β by monocytes (Iyer et al. 2009).

Accumulating evidence suggests that the Cys/CySS and GSH/GSSG redox couples can be controlled by the diet. Dietary supplementation with the cysteine precursors N-acetylcysteine or procysteine has been used extensively to counteract the effects of oxidative stress. Although the effects of these cysteine precursors usually are attributed to enhanced GSH synthesis, they also are effective even when given in combination with a GSH-synthesis inhibitor (e.g., buthionine sulfoximine) (Lailey et al. 1991). Recent studies showed that supplementing the diet with a combination of cysteine and methionine could prevent oxidation of the plasma Cys/CySS redox couple and decrease circulating levels of proinflammatory interleukin-1 β in endotoxin-challenged mice (Iyer at al 2009). Similar diets also can alter the plasma Cys/CySS redox state in humans (Jones et al. 2011). It will be interesting to determine whether this type of dietary intervention can protect against lung injury in chronic alcoholics.

Zinc deficiency, particularly within immune cells in the lungs (i.e., alveolar macrophages), also contributes to increased susceptibility to bacterial infection in chronic alcoholics (Mehta et al. 2011). Studies in rats showed that chronic alcohol feeding decreased bacterial clearance from lung and oxidized Cys/CySS in the alveolar space. Dietary zinc supplementation blocked both of these effects (Mehta et al. 2011).

### Brain Injury

Prenatal alcohol exposure can result in a range of detrimental effects, including damage to the developing brain, that are collectively known as FASD. Early autopsy studies, as well as more recent magnetic resonance imaging studies in both animal models and humans have revealed a variety of brain abnormalities, including reduced brain size (i.e., microcephaly) and anomalies of specific brain structures (e.g., the cerebrum, cerebellum, hippocampus, basal ganglia, and corpus callosum) after prenatal alcohol exposure (Lebel et al. 2011; Lipinski et al. 2012). These ethanol-induced brain insults contribute to the learning deficits, impairment in memory, difficulties with motor planning, and problems in regulating emotions and behavior observed in children with FASD.

Alcohol can damage the developing embryo through multiple mechanisms. Oxidative stress seems to play an important role in ethanol-induced programmed cell death (i.e., apoptosis) and morphological abnormalities (Chen et al. 2013). In addition, accumulating evidence suggests that changes in epigenetic regulation are involved in the pathogenesis of FASD. For example, in animal studies, prenatal alcohol exposure increased the proportion of offspring with an unusual coat color by inducing hypermethylation of a specific gene, *Avylocus (*Kaminen-Ahola et al. 2010). Moreover, recent studies demonstrated that microRNA 125b can prevent ethanol-induced apoptosis of certain embryonal cells (i.e., neural crest cells) by targeting two specific genes called *Bak1* and *PUMA* (Chen et al. 2015).

It also is well known that nutritional deficiencies contribute to the pathogenesis of FASD and to ethanol-induced damage to the developing brain. Heavy maternal alcohol consumption results in deficiency in nutrients that are critical for fetal development and maternal health, including vitamins A and D, thiamin, folate, and zinc (Dreosti 1993). Moreover, as in adult brains, DHA deficiency occurred in the developing brain of animals prenatally exposed to ethanol. Finally, studies have shown that diets low in nutrients exacerbate alcohol-induced brain damage in the offspring (Nacach et al. 2009).

Maternal nutrient supplementation may decrease the risk of FASD and serve as a potential intervention for FASD. Some nutritional interventions target oxidative stress. For example, antioxidant supplements, such as vitamins C and E, can reduce oxidative stress, cell death, and behavioral impairments in animals prenatally exposed to ethanol. Studies in the adult brain have demonstrated that ethanol-induced neuro-inflammation and degeneration can be countered by dietary DHA. Similarly, an ω-3-enriched diet that contains 24.6 percent DHA has been shown to reduce ethanol-induced oxidative stress in the developing brain (Patten et al. 2011), consistent with the relationship between dietary fat and organ injury discussed earlier. Other nutritional interventions may work through epigenetic modulations. Supplementation with nutrients that act as methyl donors, including folic acid and choline, may modulate epigenetic profiles and alter the expression of genes important for neurodevelopment. Thus, prenatal folic acid supplementation attenuated ethanol-induced malformations, growth retardation, and neuronal loss (Wang et al. 2009), whereas prenatal and postnatal supplementation with choline reduced ethanol-induced malformations and behavioral impairment (Thomas et al. 2010). Furthermore, recent studies have shown that sulforaphane, a chemical that is abundant in broccoli sprouts and which can inhibit enzymes involved in epigenetic modifications (i.e., DNA methyltransferase and histone deacetylases), can diminish ethanol-induced apoptosis in neural crest cells through induction of nuclear factor (erythroid-derived 2)-like 2 (Nrf2) (Chen et al. 2013). These findings highlight the potential of nutrient supplementation in preventing or attenuating brain damage associated with FASD, improving cognitive function in children with FASD, and attenuating brain damage in adults.

### Immune Dysfunction

Excessive alcohol consumption has deleterious effects on the immune system. Several clinical and experimental studies have suggested that long-term alcohol use can lead to the dysregulation of both cell-mediated and humoral immunity (Barve et al. 2002). Epidemiologic studies have documented that alcohol-induced impairment of the immune system leads to increased susceptibility to opportunistic infections and development of certain tumors (Barve et al. 2002). Although many types of immune cells are affected by alcohol, including neutrophils, natural killer cells, and monocytes/macrophages, several observations suggest that the major effect of ethanol involves the impairment of thymus-derived lymphocytes (T lymphocytes or T cells). Because a subgroup of T-lymphocytes (i.e., CD4+ T cells) are the central regulators of the immune system, including cell-mediated and humoral immunity, loss of their survival and function constitutes a critical part of alcohol-induced immune dysfunction.

A number of experimental animal models of ethanol abuse have established that chronic alcohol administration decreases the absolute numbers of CD4+ T cells in the thymus, spleen, lymph nodes, and periphery, as well as the immune function of these cells (Barve et al. 2002). Similarly, patients with AUD exhibit significantly reduced numbers of CD4+ T cells (Barve et al. 2002). Although other clinical complications in alcoholic patients can negatively influence the immune system, recovery of the CD4+ T-cell count was noted after alcohol withdrawal in several studies, suggesting that ethanol can directly affect CD4+ T-cell survival (Barve et al. 2002). Moreover, experimental and clinical studies have documented that alcohol intake can cause depletion of CD4+T cells, and the mechanisms underlying this effect are only beginning to be understood. Research has indicated that ethanol can potentially act as a cofactor and exacerbate clinical conditions that cause CD4+ T-cell depletion by enhancing activation-induced, fatty acid synthase–mediated apoptosis (Ghare et al. 2014). In addition to affecting CD4+ T-cell numbers, ethanol also has a major effect on T-cell function by decreasing the production of the cytokine, interleukin-2, which is critical for the clonal expansion of CD4+ T cells (Ghare et al. 2011).

In subjects with AUD, the combined effects of alcohol metabolism and compromised nutrition led to major nutrient disturbances, including deficiency of the critical nutrient metabolite, SAM. Studies found that levels of SAM as well as of methionine adenosyltransferase (MAT II), the enzyme that converts methionine to SAM, were markedly reduced in cultured CD4+ cells exposed to alcohol. This resulted in a significant upregulation of expression and activity of several enzymes involved in apoptosis, leading to increased apoptotic cell death (Hote et al. 2008). Moreover, restoration of intracellular SAM levels via SAM supplementation considerably attenuated this apoptotic death in T cells, implying a causal/protective role for SAM in T-cell survival (Hote et al. 2008).

Overall, these findings have begun to provide critical molecular insights into epigenetic mechanisms underlying the alcohol- and nutrient (SAM)-status–induced immunotoxicity in human CD4+ T cells. Because there currently is no Food and Drug Administration–approved therapy for the treatment of immune suppression associated with chronic alcohol abuse, these findings have the potential to facilitate the development of nutrient (SAM)-based therapy in alcoholic patients.

## Conclusions

Alterations in nutrition and nutrient metabolism are common in chronic alcoholics and may contribute to alcohol-induced organ injury. Conversely, nutritional supplementation may prevent the development or attenuate the progression of alcohol-induced organ injury. Nutritional supplements may alleviate a nutrient deficiency or act as pharmacologic agents. Such nutrients also may have epigenetic effects. Nutritional supplementation as a therapy is especially attractive because there are currently no Food and Drug Administration–approved therapies for most forms of alcohol-induced organ injury and nutrient supplements are readily available.

## Figures and Tables

**Figure 1:**
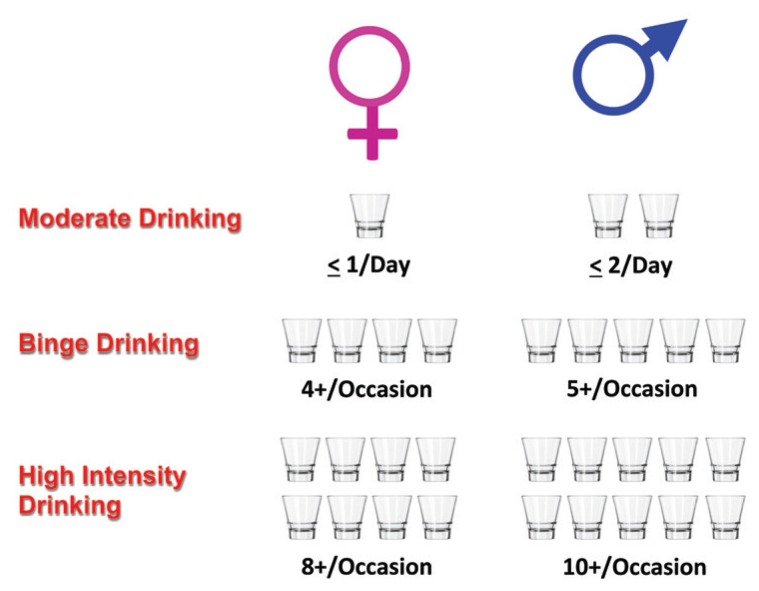
Drinking levels and their consequences. In the United States, drinking levels are expressed in terms of standard drinks consumed—that is, the number of alcoholic beverages drunk, each containing about 0.6 fluid ounce or 14 grams of pure alcohol. The *Dietary Guidelines for Americans 2015–2020* defines moderate drinking as consuming up to 2 drinks/day for men and up to 1 drink/day for women. The Substance Abuse and Mental Health Services Administration defines binge drinking as consuming 5 or more (for men) or 4 or more (for women) alcoholic drinks on the same occasion on at least 1 day in the past 30 days (National Institute on Alcohol Abuse and Alcoholism 2016). High-intensity drinking refers to drinking at levels far beyond the binge threshold, resulting in high peak blood alcohol concentrations. Some studies define high-intensity drinking as two or more times the gender-specific binge drinking thresholds (Patrick et al. 2016); others use a higher threshold (Johnston et al. 2016). Some individuals drink considerably more than this. For example, one study found that patients admitted to a National Institutes of Health treatment facility with a diagnosis of alcohol use disorder consumed the equivalent of 13 drinks per day (Vatsalaya et al. 2016). In these drinkers, the metabolic effects of alcohol and altered nutrient intake may set the stage for alcohol–nutrient interactions and organ injury.

**Table:** Types of Nutrient Deficiency Caused by Heavy Drinking and the Associated Signs and Symptoms

Selected Nutrient Deficiency	Signs/Symptoms
**Magnesium**	Insulin resistance, muscle cramps
**Selenium**	Myopathy, cardiomyopathy
**Vitamin B1/Thiamine**	Wernicke-Korsakoff syndrome, neurologic symptoms
**Vitamin B2/Riboflavin**	Glossitis, cheilitis, and lingual papillae atrophy
**Vitamin A/Retinol**	Abnormal dark adaptation, rough skin
**Vitamin C**	Scurvy with purpura and petechiae
**Vitamin D**	Altered bone metabolism, altered gut barrier/immune function
**Vitamin E**	Oxidative stress
**Niacin**	Skin photosensitivity, confusion, pellagra
**Folate, S-Adenosylmethionine**	Anemia, altered methylation, epigenetic effects

## Footnotes

1 The diet containing USFs was rich in corn oil, whereas the diet containing SFs was rich in medium-chain triglycerides.

2 The EtOH+USF-induced changes in gut microbiota were characterized by the decrease of certain bacteria (i.e., the *Bacteriodetes* phylum) with a proportional increase in others (i.e., gram-negative *Proteobacteria* and gram-positive *Actinobacteria* phyla). The bacterial genera that showed the biggest expansion were the gram-negative, alkaline-tolerant *Alcaligenes* and gram-positive *Corynebacterium* (Bull-Otterson et al. 2013).

3 This polymeric enteral solution was enriched in branched-chain amino acids, energy dense (1.3 kcal/ml), and low in fat and sodium.

## Glossary

Ascites: Accumulation of fluids in the abdominal cavity
Cardiomyopathy: A condition of the heart muscle wherein it becomes enlarged, thick, or rigid. In rare cases, the muscle tissue in the heart is replaced with scar tissue
Cell-Mediated Immunity: Part of the immune response that involves the activation of phagocytes, antigen-specific cytotoxic T-lymphocytes, and the release of various *cytokines* in response to a foreign molecule (i.e., antigen)
Cheilitis: Inflammation affecting the lips; this inflammation may include the skin around the mouth (i.e., perioral skin), the vermilion border, and/or the labial mucosa
CpG Islands: Short DNA sequences that contain high levels of the normally rare cytosine–guanine sequence among the nucleotide sequence; they are targets of *DNA methylation* and are involved in the regulation of gene transcription
Cytokines: A broad and loose category of small proteins (~5–20 kDa) that are important in cell signaling. Their release has an effect on the behavior of cells around them. They can be either proinflammatory or anti-inflammatory in their effects. DNA Methylation, Epigenetic mechanism of regulation of gene expression, in which a strand of DNA is modified by addition of a methyl group (CH_3_) to any cytosine located directly before a guanine
Encephalopathy: A syndrome of overall brain dysfunction that can have many different organic and inorganic causes
Enteral Nutrition: Delivery of nutrients in liquid form directly into the stomach or intestine
Epigenetic: Heritable or nonheritable changes in phenotype or gene expression caused by mechanisms other than changes in the underlying DNA sequence; epigenetic changes can alter the appearance and structure of the DNA or the histone proteins around which the DNA is wound (e.g., *DNA methylation, histone acetylation*), thereby influencing gene expression. Glossitis, Inflammation of the tongue
Glycogen: Large, branched carbohydrate molecule consisting of glucose residues; constitutes the major carbohydrate reserve of animals and is stored primarily in liver and muscle
Hepatorenal Syndrome: Functional kidney failure, but without pathological changes to the kidneys that is associated with cirrhosis and *ascites*
Histones: Protein structures around which DNA strands are wrapped
Histone Acetylation: *Epigenetic* modification of *histones* that involves the addition of an acetyl group
Humoral Immunity: Immunity mediated by proteins called antibodies
Interstitium: The space between cells in a tissue or organ
Metallothionein: Cysteine-rich proteins that can bind to heavy metals (e.g., zinc) through the *thiol* groups of their cysteine components. They participate in the uptake, transport, and regulation of zinc and can help control *oxidative stress*
Methionine: An essential amino acid that can supply methyl groups for various metabolic reactions
Micronutrient: Any essential dietary element required only in small quantities (e.g., trace minerals)
Myopathy: Muscular disease in which the muscle fibers do not function for any one of many reasons, resulting in muscular weakness. Oxidative Stress, An imbalance between oxidants (e.g., free radicals) and antioxidants that can lead to excessive oxidation and cell damage
Parenteral Nutrition: Intravenous administration of nutrients
Pellagra: A clinical niacin deficiency syndrome characterized by dermatitis, inflammation of the mucous membranes, diarrhea, and psychic disturbances (e.g., depression, irritability, anxiety, disorientation, or hallucinations)
Petechiae: Small, nonraised, perfectly round, purplish red spots caused by bleeding in the skin layer or beneath the mucous membranes
Purpura: Any of a group of conditions characterized by small hemorrhages in the skin, mucous membranes, or serous membranes
Redox Environment: The balance between oxidants and antioxidants in a cell or organ; often used to describe the balance of oxidized and reduced nicotinamide adenosine dinucleotide (NAD and NADH) in a biological system such as a cell or organ
S-adenosylmethionine (SAM): Common co-substrate involved in methyl group transfers, transsulfuration, and aminopropylation. Although these anabolic reactions occur throughout the body, most SAM is produced and consumed in the liver
Scurvy: Condition caused by vitamin C deficiency and characterized by weakness, anemia, spongy gums, and bleeding from the mucous membranes
Steatosis: Abnormal accumulation of lipids in the functional cells of various tissues (e.g., in the liver)
Thiol: Any organic compound containing a thiol (-SH, or sulfhydryl) group; often have strong odors resembling garlic or rotten eggs
Tight Junction: An intercellular junction between epithelial cells, at which the adjacent cell membranes are joined tightly together, forming a belt-like seal; these junctions limit the passage of small molecules and ions between cells
Zinc-Finger Protein: A protein containing a small structural motif that is characterized by the coordination of one or more zinc ions in order to stabilize the fold
